# Evidence of exhausted lymphocytes after the third anti-SARS-CoV-2 vaccine dose in cancer patients

**DOI:** 10.3389/fonc.2022.975980

**Published:** 2022-12-20

**Authors:** Javier David Benitez Fuentes, Kauzar Mohamed Mohamed, Alicia de Luna Aguilar, Carlos Jiménez García, Kissy Guevara-Hoyer, Miguel Fernandez-Arquero, M Antonia Rodríguez de la Peña, Laura Garciía Bravo, Alejandro Francisco Jiménez Ortega, Paloma Flores Navarro, Jorge Bartolome Arcilla, Bárbara Alonso Arenilla, Elvira Baos Muñoz, Alberto Delgado-Iribarren García-Campero, María Montealegre Sanz, Silvia Sanchez-Ramon, Pedro Perez Segura

**Affiliations:** ^1^ Department of Medical Oncology, Hospital Clinico San Carlos, IdISSC, Calle Profesor Martín Lagos, Madrid, Spain; ^2^ Department of Immunology, IML and IdISSC, Hospital Cliínico San Carlos, Calle Profesor Martín Lagos, Madrid, Spain; ^3^ Department of Immunology, Ophthalmology and ENT, School of Medicine, Complutense University, Madrid, Spain; ^4^ Department of Clinical Pharmacology, IdISSC, Hospital Cliínico San Carlos, Calle Profesor Martín Lagos, Madrid, Spain; ^5^ Department of Microbiology, IML and IdISSC, Hospital Cliínico San Carlos, Calle Profesor Martín Lagos, Madrid, Spain

**Keywords:** cancer, SARS-CoV-2, mRNA-1273, vaccine, booster, T cell exhaustion, T cell response, humoral response

## Abstract

**Introduction:**

Evidence is scant regarding the long-term humoral and cellular responses Q7 triggered by severe acute respiratory syndrome coronavirus 2 (SARS-CoV-2) mRNA vaccines in cancer patients after repeated booster doses. The possibility of T-cell exhaustion following these booster doses in this population has not yet been fully studied and remains uncertain.

**Methods:**

In this single-center prospective observational study, we explored the specific humoral and cellular response to S1 antigen in 36 patients with solid malignancies at baseline, and after the second and third doses of the mRNA-1273 vaccine.

**Results:**

A dual behavior was observed: 24 (66.7%) patients showed partial specific IFN-γ response after the second dose that was further enhanced after the third dose; and 11 (30.5%) already showed an optimal response after the second dose and experienced a marked fall-off of specific IFN-γ production after the third (4 patients negativization), which might suggest T cell exhaustion due to repetitive priming to the same antigen. One (2.8%) patient had persistently negative responses after all three doses. Seroconversion occurred in all patients after the second dose. We then studied circulating exhausted CD8+ T-cells in 4 patients from each of the two response patterns, those with increase and those with decrease in cellular response after the third booster. The patients with decreased cellular response after the booster had a higher expression of PD1^+^CD8^+^ and CD57^+^PD1^+^CD8^+^ exhausted T cells compared with those with an increased cellular response both *in vivo* and *in vitro*. The proportion of PD1^+^CD8^+^ and CD57^+^PD1^+^CD8^+^ exhausted T cells inversely correlated with IFN-γ production.

**Discussion:**

Our preliminary data show that the two-dose SARS-CoV-2 vaccine regimen was beneficial in all cancer patients of our study. An additional booster seems to be beneficial in suboptimal vaccine seroconverters, in contrast to maximal responders that might develop exhaustion. Our data should be interpreted with caution given the small sample size and highlight the urgent need to validate our results in other independent and larger cohorts. Altogether, our data support the relevance of immunological functional studies to personalize preventive and treatment decisions in cancer patients.

## Introduction

It has been more than two years since the occurrence of the first cases of pneumonia in Wuhan, China due to a new virus named severe acute respiratory syndrome coronavirus 2 (SARS-CoV-2) causing coronavirus disease 2019 pandemic (COVID-19) (1, 2).

Published literature suggests that adaptive immune response plays an important role in disease severity, viral clearance, and disease resolution (3, 4). It has also been shown that variants of concern can partially escape the humoral response elicited by mRNA vaccines, but not T-cell mediated response (5). Early induction of CD8^+^ T cells could account for asymptomatic disease (6). On the other hand, apoptosis-induced CD4^+^ and CD8^+^ T lymphopenia has been associated with severe COVID (7). Indeed, patients with severe COVID-19 present lymphopenia and low CD4^+^ and CD8^+^ T cells counts, as well as high percentages of programmed cell death-1 (PD-1) expression on T cells (8). Upregulation of immune checkpoint receptors, such as PD-1, appears to be also associated with disease severity, and interpreted as T-cell exhaustion (9). Nevertheless, conflicting evidence shows that PD-1 positive cells are functionally active in the acute and early convalescent phases of COVID-19, raising the question of whether PD-1 could be considered a marker of activation rather than exhaustion in COVID-19 patients, or whether PD-1 may endow different functional subsets (10, 11).

Cancer patients have been especially vulnerable to severe and life-threatening COVID-19, in addition to the disruption of their medical care during the worst periods of the pandemic (12–15). Cancer patients and other immunocompromised populations were also excluded or underrepresented in the clinical trials for the SARS-CoV-2 mRNA vaccines (16, 17).

In this framework, the efficacy of SARS-CoV-2 vaccines in immunocompromised populations is of paramount relevance for the design and implementation of vaccine strategies in these subjects. However, little is known regarding the long-term humoral and cellular responses triggered by SARS-CoV-2 mRNA vaccines in solid cancer patients after repeated booster doses. The scarce existing evidence points towards an enhanced humoral and T-cell response after the second dose (18) as well as an enhanced humoral response after an additional booster (19), although the latter seems of lower intensity compared to healthy subjects (20). Moreover, there is scant data concerning cell-mediated immunity and the potential exhaustion of T-lymphocytes in the event of repeated booster doses of SARS-CoV-2 vaccine in this population. Therefore, the question that remains unanswered is whether some patient subgroups might be benefiting from the administration of repeated boosters of SARS-CoV-2 vaccine while others might not.

In this work, we studied the specific humoral and cellular immune responses at three time points in solid cancer patients subsequently recruited. Most of the patients demonstrated an enhancement of T-cell responses after the second and third doses of the vaccine (Group 1), whereas a third of the patients showed a fall-off or even loss of T-cell response after the booster of the mRNA-1273 vaccine (Group 2). We then explored whether T cell exhaustion might explain the behavior of the second group of cancer patients.

## Methods

### Study design

This was a single-center prospective observational study. Thirty-six consecutive subjects with solid tumors under active treatment who received the standard two doses of mRNA-1273 vaccine and a booster dose were studied. No previous data on humoral or cellular immune status for SARS-CoV-2 in cancer patients were available in our center before the start of the study. During the study, samples were collected according to the visits scheduled in the care of each patient to avoid extra visits to the hospital due to the pandemic situation. Thus, the first determination was taken at baseline to evaluate prior exposure to SARS-CoV-2 and we considered a lapse period of 5 days before or after vaccination to collect the baseline sample. The second and third samples were collected 2-months after the second mRNA-1273 vaccine dose and 2-months after the third mRNA-1273 vaccine dose. The study was conducted in accordance with the guidelines of the Declaration of Helsinki. The study was reviewed and approved by the Ethics Committee of the Hospital Clínico San Carlos. Written informed consent was obtained from all individual participants included in the study.

### Evaluation of SARS-CoV-2 humoral response

Serum samples were analyzed for the detection of anti–SARS-CoV2 antibodies at the Microbiology Department at Hospital Clínico San Carlos. Antibody titers were measured using the SARS-CoV-2 IgG II Quant assay (Abbott Diagnostics) in the Alinity i equipment. The SARS-CoV-2 IgG II Quant Assay is a chemiluminescent microparticle immunoassay (CMIA) used for the qualitative and quantitative determination of IgG antibodies to SARS-CoV-2 in human serum and plasma. This assay is used to monitor the antibody response in people vaccinated against the SARS-CoV-2, by determining quantitatively IgG titers against the SARS-CoV-2 receptor-binding domain (RBD). The results were expressed as arbitrary units (AU) per milliliter. The positive threshold was 50 AU/mL following the manufacturer’s recommendation. According to EP34 Guide of CLSI (21) the ranges of results values that can be reported are 21.0-40,000 AU/mL (analytical measurement range) and 40,000-80,000 AU/mL (extended measurement range).

### Evaluation of SARS-CoV-2 cellular response

T Cell response to SARS-CoV-2 was measured using IFN-γ ELISA kit (Euroimmun, Lübeck, Germany) within 16-hours of blood withdrawal and was analyzed on a Triturus analyzer (Grifols S.A., Barcelona, Spain). Human lithium-heparin plasma, obtained after stimulation using the SARS-CoV-2 IGRA stimulation tube set (Euroimmun, Lübeck, Germany), was diluted 1:5 in the sample buffer. Afterwards, 100 μL of each calibrator (0.1-400 mUI/ml), controls and diluted samples were added to high-binding 96 well ELISA plates pre-coated with monoclonal anti-IFN-γ antibodies. After 2 hours of incubation at room temperature (RT), plates were washed 5 times with 350 μL of wash buffer. Subsequently, 100 μL of biotin-labeled anti-interferon-gamma antibody was added into each of the microplate wells and incubated for 30 minutes at RT. After following washes as described above, 100 μL peroxidase-labeled streptavidin was added and incubated for 30 minutes at RT. After five additional washes with wash buffer, 100 μL of 3,3’,5,5’-tetramethylbenzidine/peroxide (TMB/H_2_O_2_) was added to each well incubating it during 20 minutes and the absorbance was read at 450 nm after 30 minutes of adding the stop solution (sulphuric acid). The interpretation of SARS-CoV-2 IFN-γ antibody testing was as follows: <100 mUI/ml = negative, ≥100 to <200= borderline, ≥200 = positive.

### Evaluation of exhausted T-lymphocytes

Eight cancer patients and 4 healthy controls (HC) were studied for the co-expression of programmed cell death-1 (PD-1) and CD57 on CD4^+^ and CD8^+^ T cells to evaluate the presence of exhausted circulating T cells *ex vivo* and exhausted anti-S1 specific T cells *in vitro*. For the assessment of PD1 on *ex vivo* circulating T cells, blood samples were extracted in lithium-heparin blood collection tubes. We also evaluated the expression of PD-1 on *in vitro* specific anti-S1 CD T cells after stimulations with S1 protein during 24 hours. Samples were stained for 30 minutes at room temperature in the dark with monoclonal antibodies against CD3, CD4, CD8, CD57 and CD279 (PD-1) markers (BD Biosciences, USA) (**Supplementary Table S1**). After lysing with BD Pharm lyse (BD Biosciences, USA) and washing with PBS, the cells were analyzed on a BD FACSLyric flow cytometer (BD Biosciences, USA), where 100,000 cells were recorded per sample. After gating single cells and lymphocytes, T cells were identified by CD3 expression. T lymphocytes were further subdivided into CD4^+^ and CD8^+^ T cells. From CD8 T cells were identified CD57^-^PD1^+^CD8^+^, CD57^+^PD1^+^CD8^+^, CD57^-^PD1^dim^CD8^+^ and CD57^-^PD1^hi^CD8^+^ exhausted T cells subsets. These data were processed by FlowJo_V10 software. PD-1 and CD57 positivity among CD8^+^ T cells was defined based on isotype antibody control and the separation of PD1-high (PD1^hi^) from PD1^dim^ was based on mean fluorescence intensity (MFI).

### Statistical analysis

Microsoft Excel (v.14.1.0), GraphPad Prism software (version 8.1.0), and R software (version 4.0.4) were used for descriptive and statistical data analysis. Categorical variables were compared using Fisher’s exact test or chi-squared test, as appropriate. Quantitative data were analyzed with Kruskal-Wallis test or Mann–Whitney U test, as convenient. Values were expressed as means ± standard deviation (SD) or median (IQR) and p values of less than 0.05 were considered significant.

## Results

### Epidemiological characteristics of the study population

The study included 36 consecutively recruited patients (12 men and 24 women, 1:2), all of them older than 18 with solid tumors who were receiving active treatment at the outpatient facility of the Hospital Clínico San Carlos Medical Oncology Dept. Mean age was 59.36+/-9.09 years (range, 43 to 77 years). All patients received three doses of SARS-COV-2 mRNA-1273 vaccine. In the overall population, the metastatic stage disease (30.5%) was less common than the early stage disease (69.5%). The most prevalent primary tumors were breast (n=8, 22.2%]), head and neck (n=7, 19.4%), gynecologic (n=6, 16.7%) and gastrointestinal (n=5, 13.9%). Treatment protocols consisted mainly of chemotherapy (n=17, 47.3%) defined as cytotoxic drugs, immunotherapy (n=8, 22.2%) defined as antibodies that target PD-1/PD-L1, and targeted therapies (n=3, 8.3%) defined as drugs that target HER2 and EGFR, with the rest of the treatment protocols being combinations of these modalities. Patients receiving a combination of more than one treatment approach were also included: chemotherapy plus targeted therapy (n=3, 8.3%), chemotherapy plus immunotherapy (n=5, 13.9%). In the overall population, 22 patients (61%) received prior radiotherapy versus 14 patients (39%) that did not receive it. Only 1 patient (2.7%) had documented prior COVID-19 infection. Patients’ characteristics are reported in **Supplementary Table S2**.

### SARS-CoV-2 humoral response

All patients had available serologic data at baseline assessment and for the two consecutive time-points after the second and third vaccine dose, respectively. Median (IQR) IgG values at baseline was 0.0 (0.0-4.875) UA/mL, at the second analysis was 4,914 (1,458-13,906) UA/mL and at the third was 25,541 (13,215-39,083) UA/mL. The serologic results were reported as median because after exploring the dataset using a quantile-quantile plot, the data showed a non-normal distribution.

**Figure 1:**
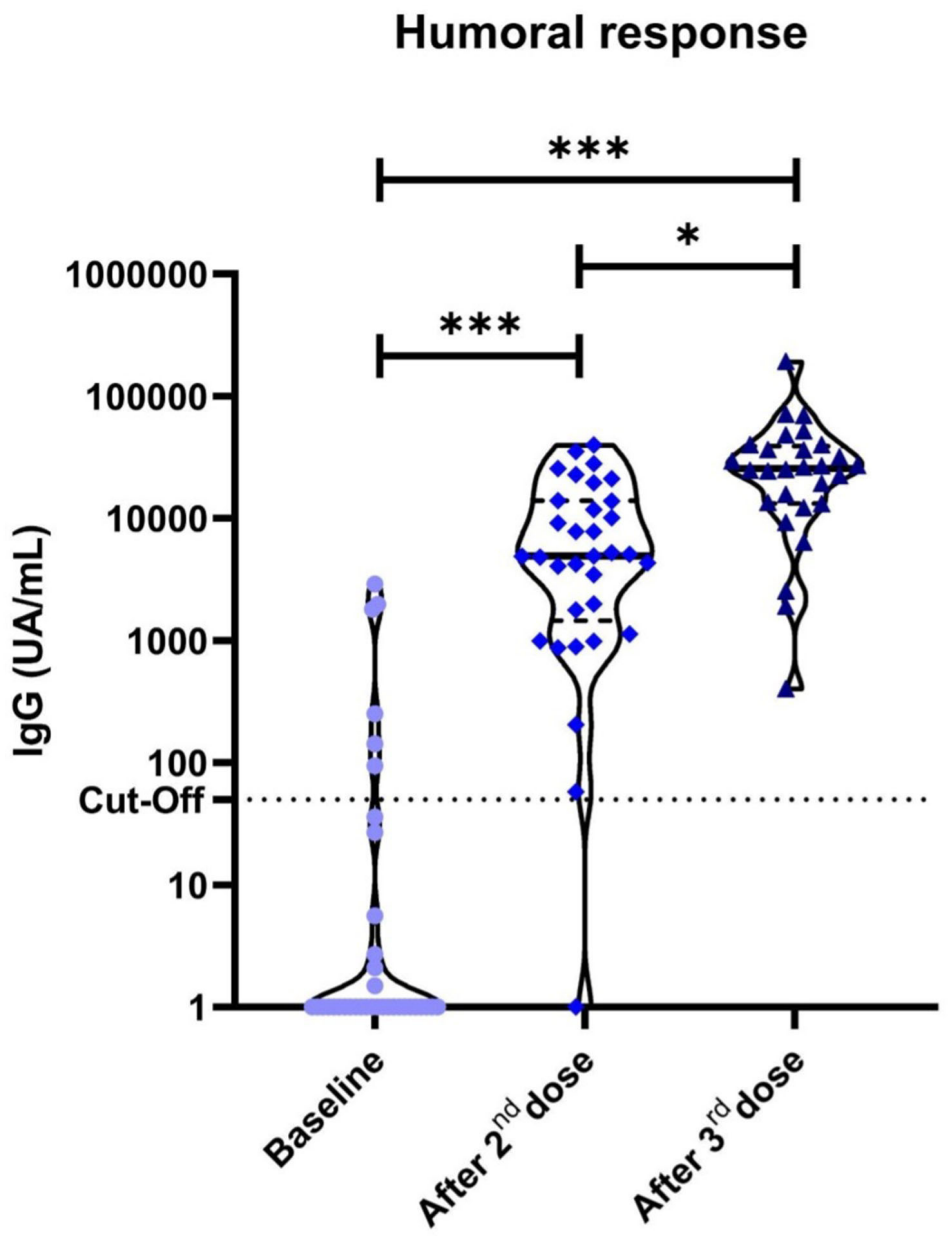
Anti-S1 IgG antibodies in cancer patients measured by chemiluminescent microparticle immunoassay. Dotted lines represent positivity cut-off: ≥50 UA/ml. Significant differences were observed in our cohort of cancer patients between the baseline anti-S1 titres and after the second (*p*<0.001) and third vaccine doses (*p*<0.001). *P<0.05 **P<0.01 ***P<0.001.

Thirty patients (83.3%) had negative IgG titers and negative cellular responses at baseline, without previous positive SARS-CoV-2 PCR or antigen tests, suggesting no prior exposure to SARS-CoV-2. Among them, serological conversion at the second evaluation occurred in all patients (100% serological conversion rate), with significantly higher titres than baseline (*p*<0.001). Significantly higher antibody titres were also seen between the first and third (*p*<0.001) and the second and third vaccine dose evaluations (*p*=0.014) (**Figure 1**).

Six (16.7%) patients were excluded from the serological conversion analysis due to positive baseline determination. Among them, 1 patient had a prior documented mild SARS-CoV-2 infection while the other 5 patients had no prior documented infection but showed low positive baseline titers of SARS-CoV-2 IgG. Median (IQR) IgG values at baseline in these 5 patients were 1,030 (143-1,810) UA/mL. In an attempt to elucidate the cause of these findings, electronic medical records were checked. All 5 patients had their serological status determination in the 5-days period after the first vaccine dose. Therefore, these results could be barely explained by the short time-lapse between vaccination and testing for baseline determination and, most probably due to secondary response.

### SARS-CoV-2 cellular response

Positive specific cellular response was displayed in 13 out of 36 (36.1%) after the first dose of the mRNA-1273 vaccine in our cancer patients, with median (IQR) IFN-γ levels of 62.25 (8-554.3) mUI/ml; while in 33 out of 36 (91.7%) after the second dose, with median (IQR) IFN-γ levels of 1,915 (690.8-1970) mUI/ml, significantly higher than baseline (*p*<0.001).

**Figure 2:**
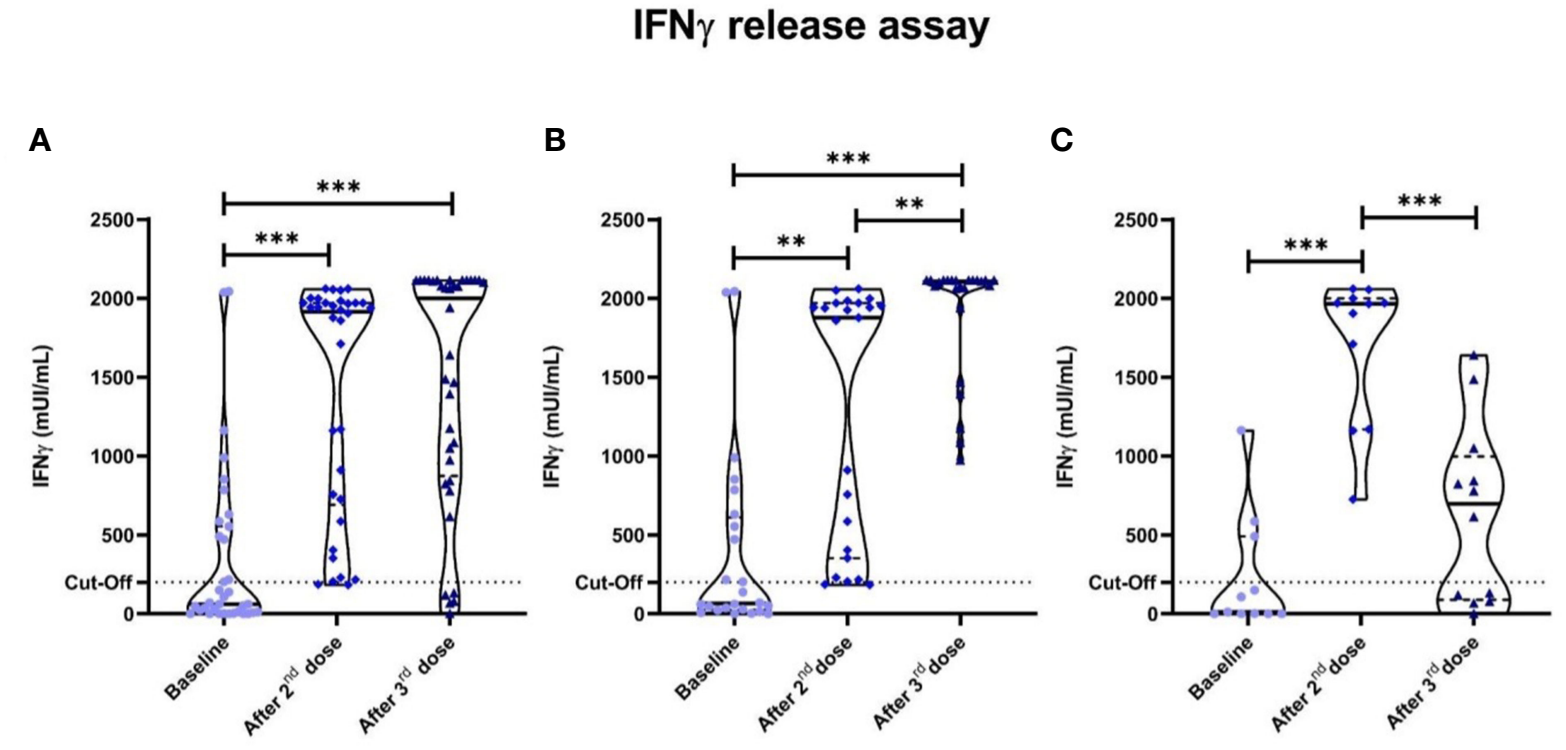
Specific anti-SARS-CoV-2 IFN-γ responses measured by IGRA. Dotted lines represent positivity cut-off: ≥200 mUI/ml. **(A)** All cancer patients. Significant differences were seen in cancer patients between the baseline anti-SARS-CoV-2 IFN-γ titres and after the second (*p*<0.001) and third vaccine doses (*p*<0.001). Two groups were established after the third dose according to the pattern of cellular behavior: one that enhanced their IFN-γ titres after the third vaccine dose (Group 1); and Group 2 that displayed a drastic fall-off of specific anti-SARS-CoV-2 IFN-γ titres. **(B)** Group 1 cancer patients. **(C)** Group 2 cancer patients. *P<0.05 **P<0.01 ***P<0.001.

After the third dose of the mRNA-1273 vaccine, we observed a dual behavior: 24 (66.7%) patients (Group 1) showed partial specific IFN-γ response after the second dose that was further enhanced after the third dose (p=0.002); while 11 (30.5%) (Group 2) already showed an optimal response after the second dose and showed a marked fall-off of specific IFN-γ production after the third (in 4 patients even negativization) (p=0.010). This later phenomenon might suggest exhaustion by repetitive priming with the same antigen (**Figure 2**). Group 1 and 2 characteristics are shown in **Supplementary Tables 3** and **Supplementary Table 4** respectively.

One remaining patient (2.8%) had a persistently negative response after all three doses. This patient was a 66 years-old female with metastatic lung cancer receiving first line chemotherapy with no prior COVID-19 infection that at the time of the third vaccine was in complete response.

Four cancer patients of 11 (36.4%) of the Group 2 displayed a negativization in specific anti-SARS-CoV-2 IFN-γ levels. Significant differences were observed between cellular responses and age in cancer patients (p=0.003). No correlations were observed either between specific IFN-γ cellular responses and gender, cancer type, treatment or patient´s tumor stage.

### T cell exhaustion

**Figure 3:**
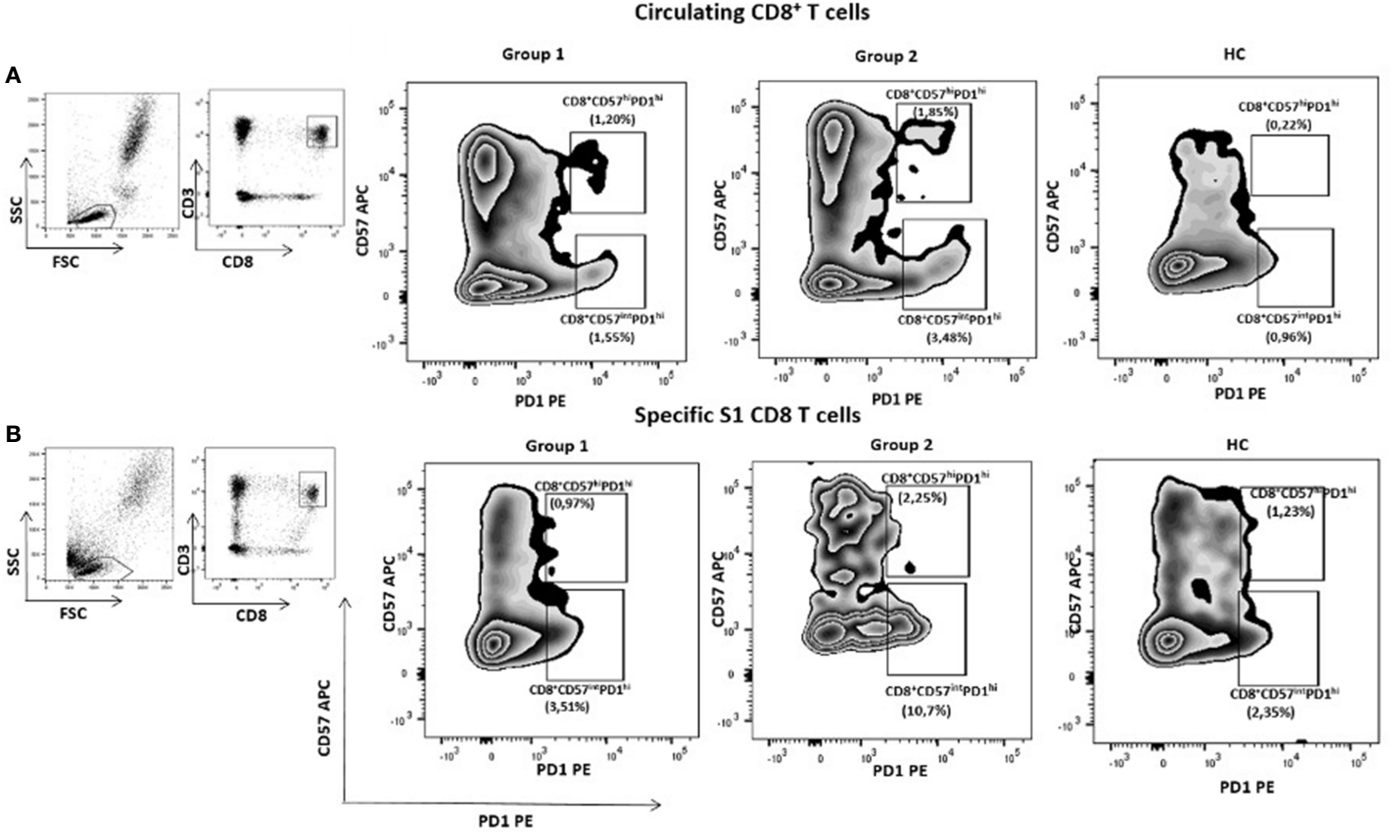
Exhausted CD8^+^ T cells according to the expression of PID and CD57 in an illustrative case of patients of each group of cancer patients and one healthy donor. All subjects have received three doses of mRNA vaccine. CD8+CD57hiPD1hi and CD8+CD57intPD1hi circulating T cells subsets were higher in group 2 compared with group 1. Similar results were seen in specific S1 CD8+ T cells.

We then sought to evaluate, after the third vaccine dose, cellular responses to SARS-CoV-2 in our cohort of cancer patients through the expression of circulating exhausted T cell markers in 4 randomly selected patients of each group and in 4 healthy controls. Besides, we assessed specific anti-S1 exhausted CD8^+^ T cells in specific proliferative tests *in vitro*. We found that the immune checkpoint PD-1 expression on CD8^+^ T cells was higher in Group 2 than in Group 1 *in vitro* and *in vivo*. Indeed, the PD1^+^CD8^+^ (p=0.06) and CD57^+^PD1^+^CD8^+^ (p=0.01) exhausted T cells subset were higher in the Group 2 compared with Group 1 and with HC *in vitro* (**Figure 3** and **Supplementary Table S5**). Interestingly, an inverse correlation was observed between the proportion of PD1^+^CD57^+^CD8^+^ T cells and IFN-γ production (r= -0.77; p=0.003), and between total PD1^+^CD8^+^ T cells and IFN-γ production (r= -0.56; p=0.05). No differences were observed after adjusting for age. Patients’ data supporting T cell exhaustion in each group are reported in **Supplementary Table S5**. No differences in CD4^+^ T cells values between the different groups were observed.

We followed our cancer patients during six months after the third dose to describe how many of them had developed COVID-19 and the clinical expression of the disease. One 66 year-old woman out of the 12 patients without adequate IFN-γ levels after the third dose presented with COVID-19, with fever up to 37.8°C, cough and malaise for 10 days, without requiring specific therapy or hospitalization. The patient had anti-SARS-CoV-2 IgG levels of 402.3 UA/mL.

## Discussion

To the best of our knowledge, this is the first evidence of exhausted CD8^+^ T cells after repeated doses of S1 antigen following SARS-CoV-2 vaccination. We evaluated in the real-life setting the cellular and humoral immune responses after each of the three doses of mRNA-1273 vaccine in a non-selected population of solid cancer patients. We found positive specific SARS-CoV-2 cellular responses in 36.1% of cancer patients after the first vaccine dose that increased to 91.7% after the second dose. Thus, our data further confirm the efficacy of the vaccine in triggering the cellular immune responses in patients with cancer in agreement with a study by Bordry et al. (18). Specific humoral responses were detected in all cancer patients after the second dose of the mRNA vaccine, in line with other studies showing that the majority of cancer patients are able to mount specific antibody responses to SARS-CoV-2 vaccines (22, 23). Exhausted CD8^+^ T cells have been also described during prolonged COVID-19 associated with PD-1 on cell surface, particularly in those patients overtly symptomatic or requiring ICU (24).

Regarding the third vaccine dose (booster), our findings differentiated two groups based on the behavior of specific cellular immune response: those who further enhanced the cellular response, which coincided with a partial response after the second dose (Group 1); and those who showed a dramatic decrease or even negativization in specific anti-SARS-CoV-2 IFN-γ titres (Group 2). In addition, one patient did not mount cellular responses to any of the three vaccine doses, despite seroconversion, suggesting T-cell independent mechanisms (25). Increased proportions of exhausted CD8^+^ T cells were observed in the Group 2 of cancer patients. Interestingly, IFN-γ production was inversely correlated with exhausted CD8^+^ T cells subsets. Our data might support the relevance of alternative preventive strategies against SARS-CoV-2 in patients with non-response to a particular vaccine platform, RNA in this particular case. In accordance with Addeo et al. (22), no differences were observed after adjusting for age, sex, type of tumor, cancer stage or the treatment patients received in IgG and/or IFN-γ levels.

A concerning phenomenon was the loss of cellular responses, which may be due, among other factors, to increased expression of T cell inhibitory molecules after the third vaccine dose, suggesting T cell exhaustion after SARS-CoV-2 vaccination with additional booster and may account for the lack of capacity of these cells to control viral replication. T cell exhaustion is a dysfunctional state of T cells characterized by the high expression level of immune-checkpoint (IC) receptors, such as PD-1 (exhaustion) and CD57 terminal effector cells (senescence) markers, decreased proliferation and production of cytotoxic cytokines, and altered transcriptional and metabolic profiles (26). These IC immune receptors have been previously demonstrated to control antiviral and antitumor CD8^+^ T-cell effector function in experimental models of lymphocytic choriomeningitis virus (LCMV) and in humans with advanced melanoma (27, 28). Madmoodpoor et al. also outlined a higher expression of PD-1 in circulating lymphocytes of patients with severe COVID-19 compared with healthy controls (8). Recurrent or latent infections by many pathogens as well as several vaccines in development have shown to induce overexpression of these IC molecules, and thus exhaustion in immune cells, leading to increases in inhibitory IC signals and immune evasion (29–31). As a consequence of increased inhibitory IC receptors, T cells are exhausted, leading to viral escape from immune control (32). However, the precise mechanisms underlying the increase in exhausted CD8^+^ T cells after SARS-CoV-2 vaccination or infection remains to be elucidated. Further functional analyses of these exhausted SARS-CoV-2-specific CD8^+^ T cells are needed to ascertain their role in the loss of protective immunity to the virus (11, 33). Another potential explanation of the loss of cellular response might be the occurrence of anti-IFN-γ antibodies described by Bastard et al. (34), which were not performed here. Importantly, the potential induction of exhausted CD8^+^ T cells should be taking into account in vaccines strategies, as suggested by results for other RNA virus vaccines such as HIV (35).

Only one of the 12 patients with low IFN-γ presented with moderate COVID-19, which could be explained by an adequate innate immunity, as has been argued for the mild manifestations in most children, or by the presence of high specific antibody titers at the short term (34). Also, specific CD4^+^ T cells clones might enhance humoral responses.

Our findings have potential implications on vaccine responses in advanced immune engaging cancer therapy patients who have particularly blunted cellular vaccine responses despite multiple doses (up to 43% of patients after the third dose of the BNT162b2 mRNA COVID-19 vaccine) (36). Biomarkers that predict SARS-CoV-2 vaccine response remain to be determined in profoundly immunosuppressed patients’ populations, such as allogeneic hematopoietic cell transplant (HCT) and chimeric antigen receptor T cell (CAR-T) therapy recipients, which have high susceptibility to infections, and of severe COVID-19 (37). Our results on the induction of exhausted CD8^+^ T cells by booster doses in a subgroup of patients are particularly relevant in CAR-T recipients, in which exhausted CD8^+^ T-cells seem to play a relevant role in the lack of response in B cell malignancies as well as in the loss of effectiveness of the CAR T cells (38). In this regard, emerging data shows that vaccination against SARS-CoV-2 in recipients of CAR T-cell therapy render a low rate of seroconversion (30%) even though third and fourth vaccine doses indicating the need for additional infection-vaccination strategies (39–41). Combined humoral and cellular anti-SARS-CoV-2 responses may prevent severe disease in HCT and CART-T recipients (37). New evidence also suggests that, among patients receiving CAR-T therapies, some may respond better to vaccines than others (41, 42). Therefore, it is critical to understand the molecular and cellular mechanisms underlying the polarization towards exhausted T cell responses after vaccine boosters in these immunosuppressed individuals.

This study has several limitations that should be mentioned and thus the results interpreted with caution, regarding its relatively small sample size and the heterogeneity of the cancer patients. One potential limitation regarding the baseline determination is the 5-day period pre and post vaccine established to collect the first sample that seems to slightly affect the baseline results in 5 patients of the study. Additionally, the IGRA test does not differentiate between CD4^+^ and CD8^+^ SARS-CoV-2-specific T cells. Further studies are needed to better understand the degree of each cell subset participation in the response after infection or vaccination. Therefore, we present the work as a pilot exploratory analysis.

To summarize, our preliminary study shows that most of our cancer patients develop cellular and humoral responses after two-dose SARS-CoV-2 vaccines. The third vaccine dose seems to be beneficial in non-optimal responders to SARS-CoV-2 vaccines who displayed an increase in anti-SARS-CoV-2 IFN-γ titres. Nevertheless, maximal responders might develop exhaustion by persistent antigen stimulation, which is of important concern in this patient population. Based on our data, we believe in the necessity of these functional immunological studies to better define the vaccination strategies for cancer patients.

## Data availability statement

The raw data supporting the conclusions of this article will be made available by the authors, without undue reservation.

## Ethics statement

The studies involving human participants were reviewed and approved by Ethics Committee of Hospital Clínico San Carlos. The patients/participants provided their written informed consent to participate in this study.

## Author contributions

JDBF, KM, AL, SS-R and PP conceived of and designed the study. JDBF, KM, and AL performed the literature search. CJ, JDBF, AL and BA generated the figures and tables. JDBF, KM and AL analyzed the data and wrote the manuscript. KG and CJ performed the statistical analysis. SS-R and PP wrote specific parts of the article and critically reviewed the manuscript and supervised the research. All authors contributed to the article and approved the submitted version.
