# Supplementary material for: Evidence of exhausted lymphocytes after the third anti-SARS-CoV-2 vaccine dose in cancer patients
Source: Front Oncol. 2022 Dec 20;12:975980. doi: 10.3389/fonc.2022.975980 (PMC9808030; doi:10.3389/fonc.2022.975980)
Supplement: Supplementary file 1 [file Table_1.docx]

| **Specificity** | **Fluorophore** | **Target clone** |
| --- | --- | --- |
| CD3 | BV605 | HIT3A |
| CD4 | BV510 | RM4-5 |
| CD8 | APC-H7 | SK1 |
| CD57 | APC | NK-1 |
| CD279 (PD-1) | PE-CY7 | EH12.1 |

**Supplementary Table 1.** Reagents used on the exhausted CD8^+^ T cell panel.
