# Supplementary material for: Evidence of exhausted lymphocytes after the third anti-SARS-CoV-2 vaccine dose in cancer patients
Source: Front Oncol. 2022 Dec 20;12:975980. doi: 10.3389/fonc.2022.975980 (PMC9808030; doi:10.3389/fonc.2022.975980)
Supplement: Supplementary file 2 [file Table_2.docx]

| **Characteristic** | **No. (%)** |
| --- | --- |
| **Total population** | 36 (100) |
| Mean age, y | 59,36 |
| Sex |  |
| Male | 12 (33.3) |
| Female | 24 (66.7) |
| Primary diagnosis |  |
| Breast | 8 (22.2) |
| Gastrointestinal | 5 (13.9) |
| Lung | 4 (11.1) |
| Genitourinary | 2 (5.6) |
| Gynecologic | 6 (16.7) |
| Head and neck | 7 (19.4) |
| Others | 4 (11.1) |
| Stage (%) |  |
| I | 4 (11.1) |
| II | 8 (22.2) |
| III | 10 (27.8) |
| IV | 14 (38.9) |
| Stage |  |
| Local | 25 (69.5) |
| Metastatic | 11 (30.5) |
| Treatment |  |
| Chemotherapy | 17 (47.3) |
| Immunotherapy | 8 (22.2) |
| Targeted therapy | 3 (8.3) |
| Chemotherapy plus targeted therapy | 3 (8.3) |
| Chemotherapy plus immunotherapy | 5 (13.9) |
| Immunotherapy plus targeted therapy | 0 (0) |
| Chemotherapy plus Immunotherapy plus targeted therapy | 0 (0) |
| Prior/concomitant Radiotherapy |  |
| Yes | 22 (61) |
| No | 14 (39) |
| Prior COVID-19 infection |  |
| Yes | 1 (2.7) |
| No | 35 (97.3) |

**Supplementary Table 2.** Baseline characteristics of the patients at the beginning of the study.
