# Supplementary material for: Evidence of exhausted lymphocytes after the third anti-SARS-CoV-2 vaccine dose in cancer patients
Source: Front Oncol. 2022 Dec 20;12:975980. doi: 10.3389/fonc.2022.975980 (PMC9808030; doi:10.3389/fonc.2022.975980)
Supplement: Supplementary file 3 [file Table_3.docx]

| **Characteristic** | **No. (%)** |
| --- | --- |
| **Total population** | 24 (100) |
| Mean age, years | 56.2 |
| Sex (%) |  |
| Male | 8 (33.3) |
| Female | 16 (66.7) |
| Primary diagnosis (%) |  |
| Breast | 6 (25) |
| Gastrointestinal | 2 (8.3) |
| Lung | 2 (8.3) |
| Genitourinary | 2 (8.3) |
| Gynecologic | 5 (20.8) |
| Head and neck | 4 (16.7) |
| Others | 3 (12.6) |
| Stage (%) |  |
| I | 4 (16.7) |
| II | 5 (20.8) |
| III | 7 (29.2) |
| IV | 8 (33.3) |
| Stage at the beginning of the study (%) |  |
| Local | 19 (79.2) |
| Metastatic | 5 (20.8) |
| Stage at the time of third vaccine dose (%) |  |
| Local | 11 (45.8) |
| Metastatic | 13 (54.2) |
| Assessment of treatment response at the time of third vaccine dose (%) |  |
| No evidence of disease | 3 (12.5) |
| Complete response | 8 (33.3) |
| Stable disease | 4 (16.7) |
| Partial response | 3 (12.5) |
| Progressive disease | 6 (25) |
| Treatment (%) |  |
| Chemotherapy | 12 (50) |
| Immunotherapy | 6 (25) |
| Targeted therapy | 2 (8.3) |
| Chemotherapy plus targeted therapy | 0 (0) |
| Chemotherapy plus immunotherapy | 4 (16.7) |
| Immunotherapy plus targeted therapy | 0 (0) |
| Chemotherapy plus Immunotherapy plus targeted therapy | 0 (0) |
| Heavily treated (three or more lines of treatment) at the time of third vaccine dose (%) |  |
| Yes | 9 (37.5) |
| No | 15 (63.5) |
| Prior/concomitant Radiotherapy (%) |  |
| Yes | 17 (70.8) |
| N | 7 (29.2) |
| Prior COVID-19 infection (%) |  |
| Yes | 1 (4.2) |
| No | 23 (95.8) |

**Supplementary Table 3.** Group 1 patient characteristics at third dose administration
