# Supplementary material for: Evidence of exhausted lymphocytes after the third anti-SARS-CoV-2 vaccine dose in cancer patients
Source: Front Oncol. 2022 Dec 20;12:975980. doi: 10.3389/fonc.2022.975980 (PMC9808030; doi:10.3389/fonc.2022.975980)
Supplement: Supplementary file 4 [file Table_4.docx]

| **Characteristic** | **No. (%)** |
| --- | --- |
| **Total population** | 11 (100) |
| Mean age, y | 65.5 |
| Sex (%) |  |
| Male | 4 (36.4) |
| Female | 7 (63.6) |
| Primary cancer diagnosis (%) |  |
| Breast | 2 (18.2) |
| Gastrointestinal | 3 (27.3) |
| Lung | 1 (9.1) |
| Genitourinary | 0 (0) |
| Gynecologic | 1 (9.1) |
| Head and neck | 3 (27.3) |
| Others | 1 (9.1) |
| Stage (%) |  |
| II | 3 (27.3) |
| III | 3 (27.3) |
| IV | 5 (45.4) |
| Stage at the beginning of the study (%) |  |
| Local | 6 (54.5) |
| Metastatic | 5 (45.5) |
| Stage at the time of third vaccine dose (%) |  |
| Local | 2 (18.2) |
| Metastatic | 9 (81.8) |
| Assessment of treatment response at the time of third vaccine dose (%) |  |
| No evidence of disease | 2 (18.2) |
| Complete response | 2 (18.2) |
| Stable disease | 0 (0) |
| Partial response | 2 (18.2) |
| Progressive disease | 5 (45.5) |
| Treatment (%) |  |
| Chemotherapy | 4 (36.4) |
| Immunotherapy | 2 (18.2) |
| Targeted therapy | 1 (9.1) |
| Chemotherapy plus targeted therapy | 3 (27.3) |
| Chemotherapy plus immunotherapy | 1 (9.1) |
| Immunotherapy plus targeted therapy | 0 (0) |
| Chemotherapy plus Immunotherapy plus targeted therapy | 0 (0) |
| Heavily treated (three or more lines of treatment) at the time of third vaccine dose (%) |  |
| Yes | 4 (36.4) |
| No | 7 (63.6) |
| Prior/concomitant Radiotherapy (%) |  |
| Yes | 4 (36.4) |
| No | 7 (63.6) |
| Prior COVID-19 infection (%) |  |
| Yes | 0 (0) |
| No | 11 (100) |

**Supplementary Table 4.** Group 2 patients characteristics at third dose administration.
