# Supplementary material for: Evidence of exhausted lymphocytes after the third anti-SARS-CoV-2 vaccine dose in cancer patients
Source: Front Oncol. 2022 Dec 20;12:975980. doi: 10.3389/fonc.2022.975980 (PMC9808030; doi:10.3389/fonc.2022.975980)
Supplement: Supplementary file 5 [file Table_5.docx]

|  | **Group 1** | **Group 2** | ***P*** |
| --- | --- | --- | --- |
| % PD1^+^CD8^+^ T cells | 7.1 (3.1-9.7) | 19.5 (14.6-29.9) | *0.06* |
| % PD1^dim+^CD57^-^CD8^+^ T cells | 3.4 (0.8-6.4) | 8.2 (4.3-19.9) | *0.19* |
| % PD1^hi+^CD57^-^CD8^+^ T cells | 0.1 (0-0.3) | 0.27 (0.1-1.4) | *0.19* |
| % PD1^+^CD57^+^CD8^+^ T cells | 2.8 (2.4-4.6) | 11.7 (9.1-14.1) | *0.01* |

**Supplementary Table 5.** Exhausted CD8^+^ T cells according to expression of PID and CD57 in both groups of cancer patients. Expressed in median values (IQR), as proportions of total CD8^+^ T cells. Comparisons were performed by using Wilcoxon rank sum exact test.
